# Efficacy and safety of acupuncture for senile insomnia: A protocol for systematic review and meta-analysis

**DOI:** 10.1097/MD.0000000000026700

**Published:** 2021-07-23

**Authors:** Jiuru Wu, Yan Shi, Xichen Wang, Xiukun Dong, Xinyu Liu, Guojian Wang, Yinghua Hu

**Affiliations:** a Department of Acupuncture and Tuina, Changchun University of Chinese Medicine, Changchun, China; b Department of Acupuncture and Tuina, Affiliated Hangzhou First People's Hospital, Zhejiang University School of Medicine, Hangzhou, China; c School of Medical Information, Changchun University of Chinese Medicine, Changchun, China; d Department of Quality Management, Medical records room, Tuberculosis Hospital of Jilin Province, ChangChun, China; e Department of Chinese Medicine, Hong Kong Baptist University, Kowloon Tong, Hong Kong, China; f Department of Acupuncture and Moxibustion, Beijing Daxing District People's Hospital, Beijing, China.

**Keywords:** acupuncture, meta-analysis, senile insomnia

## Abstract

**Background:**

Senile insomnia seriously affects the quality of life of the elderly. With the increase of the proportion of insomnia in the elderly, compared with the elderly with normal sleep quality, the elderly with long-term insomnia are more likely to have dizziness, fatigue, and decreased immunity. Acupuncture has shown good effects in the treatment of insomnia. At present, there is a lack of systematic review on acupuncture in the treatment of senile insomnia. We conduct this study to evaluate the efficacy and safety of acupuncture in the treatment of senile insomnia.

**Methods:**

We will search Chinese and English databases: China National Knowledge Infrastructure, Chinese Scientific and Journal Database, Wan Fang database (Wanfang), Chinese Biomedical Literature Database, PubMed, EMBASE, Cochrane library to identify articles of randomized clinical trials of acupuncture for senile insomnia. All above electronic databases will be searched from inception to September 1, 2021. RevMan 5.3 software will be used to conduct this systematic review.

**Results:**

The study will prove the efficacy and safety of acupuncture for senile insomnia.

**Conclusion:**

We plan to submit this systematic review to a peer-reviewed journal.

**INPLASY registration number:**

INPLASY202160106.

## 1 Introduction

Insomnia is a common disease in the elderly. It is mainly characterized by difficulty falling asleep and sleep maintenance disorder, the incidence rate of which gradually increases with age.^[1,2]^ Studies have shown that: 50% of the elderly were troubled by insomnia,^[3–5]^ the main reason of which is that the growth of age leads to the degeneration of the central nervous system, which leads to the disturbance of sleep rhythm at night. Insomnia can also cause psychological diseases and physical diseases, which seriously affect the quality of life of the elderly the next day. At present, drugs are mainly used for the treatment of senile insomnia patients, but the clinical effect is not good, at the same time there are more adverse reactions, long-term using of drugs can also produce drug dependence easily. Non-benzodiazepines, which are widely used in the treatment of senile insomnia, have strong adverse reactions such as drug tolerance, addiction, and withdrawal.^[6]^ Studies showed that acupuncture in the treatment of insomnia has the advantages of good curative effect, fewer adverse reactions, simple operation, and so on.^[7–9]^ To evaluate the efficacy and safety of acupuncture in the treatment of senile insomnia, we conducted this systematic review and meta-analysis on the published randomized clinical trials (RCTs) of acupuncture for senile insomnia. This study may provide a scientific reference basis for the treatment of senile insomnia.

## 2 Methods

### 2.1 Inclusion criteria

Patients (≥60 years) with senile insomnia regardless of any race and gender; RCTs of acupuncture for senile insomnia which were published in English and Chinese will be included; in the treatment group, acupuncture was used alone or combined with other therapies, while in the control group, acupuncture was not included; primary outcome: Pittsburgh sleep quality index, the insomnia severity index.

### 2.2 Exclusion criteria

The data in the article is incomplete or incorrect, which cannot be analyzed. One of the repeated articles will be selected. The study population was senile insomnia with other diseases or serious complications. The same acupuncture therapy was used in the experimental group and the control group. The purpose of the study was not to verify the effectiveness of acupuncture.

### 2.3 Search strategies

We will search Chinese and English databases: China National Knowledge Infrastructure, Chinese Scientific and Journal Database, Wan Fang database (Wan fang), Chinese Biomedical Literature Database, PubMed, EMBASE, Cochrane library to identify articles of RCTs of acupuncture for senile insomnia. Take PubMed as an example: the key words include senile insomnia, needle, acupunture, acupunture therapy, and so on. All above electronic databases will be searched from inception to September 1, 2021.

### 2.4 Literature screening and data extraction

#### 2.4.1 Literature screening

Two researchers (XW and XD) will independently screen the literature according to the inclusion and exclusion criteria, then they will cross-check the data which was extracted, and the disagreement will be solved by discussing within them. They will first read the title and abstract. Then they will further read the full text to determine whether the studies could be included.

**Figure 1:**
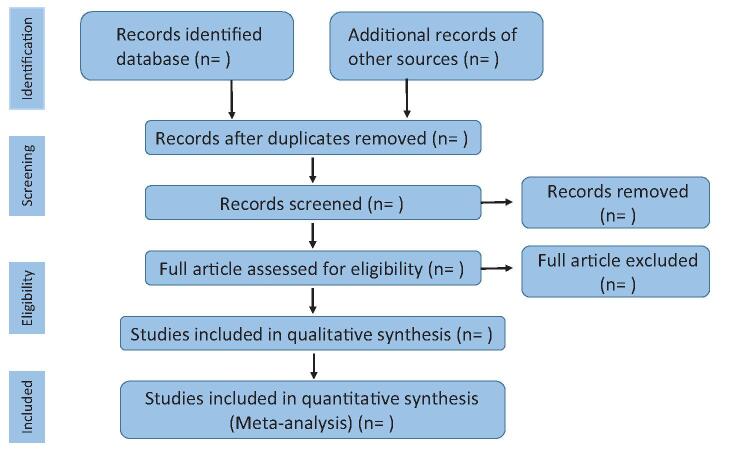
The screening process.

The process of literature screening: using Endnote software to eliminate duplicate literature; reading the title and abstract of the article, the irrelevant literature will be excluded; read the full text of the remaining articles to determine whether the studies will be included in the study (Fig. 1 the screening process).

#### 2.4.2 Data extraction

The following information will be extracted: characteristics of research: title, authors, research year, and country; basic characteristics of research objects: number of cases, intervention measures, types of treatment, intervention time, outcome indicators, etc; the key elements of bias risk assessment; data of outcomes.

### 2.5 Assessment of risk of bias in included studies

Two researchers (YH and JW) will evaluate the bias risk of the included studies with the risk assessment tool of RCT bias recommended by Cochran Collaboration Network Bias Risk Assessment Tool. When disagreements arise, they would seek a third researcher to negotiate.

### 2.6 Assessment of heterogeneity and reporting bias

We will use the Standard *I*^2^ test to assess the statistical heterogeneity, *I*^2^ < 50% indicates insignificant, when *I*^2^ ≥ 50% indicates significant. We will use a funnel plot to assess the reporting bias.

### 2.7 Statistical analysis of data

We will use Revman 5.3 software for meta-analysis. We will use relative risk as the effective index for the count data, mean difference will be used for the measurement data as the effective index. The confidence interval of each effect index was set to 95%. At the same time, the heterogeneity will be quantitatively assessed with *I*^2^. If there was no statistical heterogeneity among the studies, the fixed-effect model will be used for meta-analysis. If it is heterogeneous, the random-effects model will be used. *P* < .05 indicates statistical significance.

### 2.8 Analysis of subgroups or subsets

When there is some potential heterogeneity in this study, we may conduct subgroup analysis according to the genders, different ages, and different treatment times of included participants, if all the information could be available from included studies.

### 2.9 Sensitivity analysis

Sensitivity analysis will be conducted for assessing the robustness of the included results. Studies of high-risk bias will be excluded if the results are unstable.

### 2.10 Ethics and publication

There is no need of ethical approval because no data will be collected from individual people. We will submit the result of this study to a peer-reviewed journal for publishing.

## 3 Discussion

Acupuncture is a treatment method of Traditional Chinese Medicine, which is recommended for the treatment of 43 diseases by WHO in 1979. Acupuncture is widely used for insomnia all over the world.^[10–13]^ At present, the literature on acupuncture treatment of insomnia is increasing year by year.^[7,9,14,15]^ Senile insomnia is a common disease of the elderly, which has been attracting more and more attention, because of the increasing incidence rate in many countries. In recent years, lots of RCTs of acupuncture for senile insomnia have been conducted, but there is still a lack of systematic review on the effectiveness and safety of acupuncture in the treatment of senile insomnia, which is not conducive to the promotion and application of acupuncture in the treatment of senile insomnia. We hope that this study can provide high-level evidence for the application of acupuncture in the treatment of senile insomnia.

## Author contributions

Yinghua Hu is the designer of the article. Jiuru Wu and Guojian Wang drafted this manuscript. Yan Shi and Xinyu Liu developed the search strategy. Xichen Wang and Xiukun Dong were independently screened the potential studies and extracted data. Yinghua Hu and Jiuru Wu were assessed the risk of bias and finished data synthesis. Yinghua Hu conducted a final review of the research scheme to ensure that the scheme was correctly expressed. All authors reviewed and approved the final version of the study protocol.

**Conceptualization:** Yinghua Hu, Jiuru Wu.

**Data curation:** Xichen Wang, Xiukun Dong, Guojian Wang, Yan Shi.

**Formal analysis**: Xiukun Dong, Guojian Wang, Xinyu Liu.

**Funding acquisition:** Yinghua Hu.

**Methodology:** Xinyu Liu, Yan Shi.

**Resources**: Yinghua Hu.

**Software:** Guojian Wang, Yan Shi.

**Writing – original draft:** Jiuru Wu, Yan Shi.

**Writing – review & editing:** Yinghua Hu.
